# Impaired immunity and high attack rates caused by SARS‐CoV‐2 variants among vaccinated long‐term care facility residents

**DOI:** 10.1002/iid3.679

**Published:** 2022-08-17

**Authors:** Dorothée Obach, Anna Solastie, Oona Liedes, Saimi Vara, Eva Krzyżewska‐Dudek, Luise Brinkmann, Anu Haveri, Charlotte C. Hammer, Timothée Dub, Seppo Meri, Tobias L. Freitag, Outi Lyytikäinen, Merit Melin

**Affiliations:** ^1^ Department of Health Security, Infectious Disease Control and Vaccinations Unit Finnish Institute for Health and Welfare Helsinki Finland; ^2^ European Program for Intervention Epidemiology Training (EPIET) European Centre for Disease Prevention and Control, (ECDC) Stockholm Sweden; ^3^ Department of Health Security, Expert Microbiology Unit Finnish Institute for Health and Welfare Helsinki Finland; ^4^ Department of Bacteriology and Immunology, Translational Immunology Research Program University of Helsinki Finland; ^5^ Hirszfeld Institute of Immunology and Experimental Therapy, Department of Immunology of Infectious Diseases Polish Academy of Sciences Wroclaw Poland

**Keywords:** cell‐mediated immunity, COVID‐19, elderly, long‐term care facility, neutralizing antibodies

## Abstract

**Introduction:**

Long‐term care facilities (LTCF) residents are at high risk for severe coronavirus disease 2019 (COVID‐19), and therefore, COVID‐19 vaccinations were prioritized for residents and personnel in Finland at the beginning of 2021.

**Methods:**

We investigated COVID‐19 outbreaks in two LTCFs, where residents were once or twice vaccinated. After the outbreaks we measured immunoglobulin G (IgG) antibodies to severe acute respiratory syndrome coronavirus 2 spike glycoprotein, neutralizing antibody (NAb) titers, and cell‐mediated immunity markers from residents and healthcare workers (HCWs).

**Results:**

In LTFC‐1, the outbreak was caused by an Alpha variant (B.1.1.7) and the attack rate (AR) among once vaccinated residents was 23%. In LTCF‐2 the outbreak was caused by a Beta variant (B.1.351). Its AR was 47% although all residents had received their second dose 1 month before the outbreak. We observed that vaccination had induced lower IgG concentrations, NAb titers and cell‐mediated immune responses in residents compared to HCWs. Only 1/8 residents had NAb to the Beta variant after two vaccine doses.

**Conclusions:**

The vaccinated elderly remain susceptible to breakthrough infections caused by Alpha and Beta variants. The weaker vaccine response in the elderly needs to be addressed in vaccination protocols, while new variants capable of evading vaccine‐induced immunity continue to emerge.

## Graphical abstract

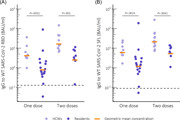

## 1 INTRODUCTION

Elderly residents in long‐term care facilities (LTCFs) have been at the highest risk of severe coronavirus disease (COVID‐19) and a fatal outcome. In 2020, COVID‐19 case fatality rate in European LTCFs has ranged between 12% and 70%.^1^, ^2^, ^3^, ^4^ Thus, the elderly have been prioritized in the severe acute respiratory syndrome coronavirus 2 (SARS‐CoV‐2) vaccine programs. In early 2021 Finland began offering COVID‐19 vaccines to LTCF residents and healthcare workers (HCW) to reduce SARS‐CoV‐2 infections in people at the highest risk of severe illness and death.

However, the antibody response to COVID‐19 vaccination is age‐dependent with lower levels of antibodies induced in subjects aged 65 and older.^5^, ^6^, ^7^ Furthermore, some variants of concern can escape vaccine‐mediated immunity.^5^, ^8^, ^9^, ^10^ Suboptimal immunity and the emergence of variants affect the risk for severe disease in real‐life conditions in the elderly, but the extent of this has been insufficiently investigated.

After the introduction of SARS‐CoV‐2 vaccination to LTCF residents and personnel, COVID‐19 outbreaks caused by the Alpha (B.1.1.7) and Beta (B.1.351) variants occurred in two LTCFs in the Helsinki Metropolitan area. We investigated the extent of the outbreaks and analysed the immune responses to vaccines and SARS‐CoV‐2 infection by measuring neutralizing antibody (NAb) titers. In addition, we measured immunoglobulin G (IgG) levels to SARS‐CoV‐2 spike glycoprotein and cell‐mediated immune responses.

## 2 METHODS

### 2.1 Setting and description of the outbreaks

In LTCF‐1, the first PCR‐positive case was diagnosed the 31 January and the last known case the 13 February 2021. All residents lived in individual rooms with bathrooms so hence room quarantine was possible. A total of 31 HCWs cared for the residents. LTCF‐2 consisted of four different floors, and the outbreak was limited to the fourth floor. A total of 34 personnel were identified of whom 30 worked on the fourth floor. The first PCR‐positive case was diagnosed the 27 February and the last known case the 25 March 2021.

### 2.2 Field visits and data collection

We retrieved information on residents and HCWs using questionnaires and residents' medical files and collected nasopharyngeal swabs and sera from the residents and HCWs willing to participate. We visited LTCF‐1 on the 24 and 25 February 2021. We visited LTCF‐2 on the 6, 8 and 21 April 2021. We assessed risk factors for the residents as either present or absent from the notes in the nursing files, and from the interviews for the HCWs. We collected demographic data, as data on comorbidities associated with the disease. Vaccination was investigated with three levels: vaccinated twice, vaccinated once, not vaccinated. The dates and results of PCR were retrieved from information given by the local epidemiological teams.

### 2.3 Case and exposition definitions

Depending on the laboratory findings, we considered two following case definitions for our analyses. The narrow case definition (PCR+) in what a case of COVID‐19 was defined as a HCW or a resident of the LTCFs with a positive PCR‐test for SARS‐CoV‐2 between the end of January and the end of February for the LTCF‐1, and between mid‐February 2021 and end of March 2021 for the LTCF‐2. The extended case definition was only applied to the unvaccinated participants. In the extended case definition, in addition to the narrow case definition, a COVID‐19 case was defined as a HCW with NAb (titer >4) to SARS‐CoV‐2 Alpha or Beta variant (depending on the outbreak) and a negative PCR‐test. Residents and HCWs were considered exposed if they were at the LTCF1, or on the 4th floor of LTFC2 during the same respective timing described above.

### 2.4 Fluorescent multiplex immunoassay

We used an in‐house fluorescent multiplex immunoassay for the measurement of IgG antibodies to WT SARS‐CoV‐2 full‐length spike glycoprotein (SFL) and receptor binding domain (RBD) as previously described.^11^

### 2.5 Microneutralization test

To measure NAb to SARS‐CoV‐2, we used the cytopathic effect‐based microneutralization test as previously described.^11^, ^12^, ^13^ NAb titers were measured to viral isolates representative of WT B lineage (hCoV‐19/Finland/1/2020, GISAID accession ID EPI_ISL_407079; GenBank accession ID MZ934691), Alpha B.1.1.7 lineage (hCoV‐19/Finland/THL‐202102301/2021, EPI_ISL_2590786; MZ944886), and Beta B.1.351 lineage (hCoV‐19/Finland/THL‐202101018/2021, EPI_ISL_3471851; MZ944846) variants. NAb titer for the World Health Organization international standard^14^ for SARS‐CoV‐2 antibody assays measured at 192, 96, and 8 to WT virus, Alpha and Beta variant, respectively. Samples with a NAb titer of <4 were considered negative and given an arbitrary titer of 2 for statistical analyses. Samples with a titer of 4 were considered borderline‐positive, and samples with a titer of >4 positive.

### 2.6 Cell isolation and stimulation

Peripheral blood mononuclear cells  (PBMCs) were collected using BD Vacutainer® CPT™ Mononuclear Cell Preparation Tubes (BD 362760) from HCWs (*n* = 3) and residents (*n* = 9) in LTCF‐2. PBMCs were isolated from CPT™ tubes and cells were washed with Ficoll salt solution. CryoStor® CS10 was added to the cell samples and the samples were stored in liquid nitrogen for further analysis.

### 2.7 Human PBMC stimulation assay

Functional responses by PBMC were tested, using a published protocol.^15^ Briefly, PBMCs were stimulated for 6 days in RPMI 1640 culture medium (Life Technologies) supplemented with 5% of heat‐inactivated Human AB serum (Innovative Research), with 2 µg/ml anti‐CD3/anti‐CD28 antibody (BioLegend; positive control), medium (negative control), 10 µg/ml SARS‐CoV‐2 recombinant nucleoprotein (Leinco Technologies) or spike glycoprotein (a gift from Dr. Olli Ritvos, University of Helsinki) expressed in HEK293 cells.

### 2.8 Fluorescent multiplex bead assay for quantification of T cell cytokines and effector molecules

Interferon gamma  (IFN‐γ), granzyme B and perforin‐1 in supernatants from human PBMC stimulation assays were measured in 96‐well plates using the MILLIPLEX® MAP Kit HCD8MAG‐15K from Millipore®, according to manufacturer instructions with Luminex MAGPIX magnetic bead analyzer (Luminex®Corporation). Median fluorescent intensity was converted to concentration by interpolation from 7 diluted standards using a 5‐parameter logistic regression. Concentrations were further converted into stimulation indexes from the concentration of the negative control. Samples below the lowest standard in the linear range were given half the value of this standard (0.0024 ng/ml for IFN‐γ, 0.0006 ng/ml for granzyme B, 0.02 ng/ml for perforin‐1). Sample values exceeding the highest standard in the linear range were given the value of this standard (5 ng/ml for IFN‐γ and granzyme B, 50 ng/ml for perforin‐1). Standards with a standard deviation of less than 20% for the duplicates were accepted. Samples measured with <35 beads/antigen/well were excluded from the analysis. The stimulation index threshold for a positive result was ≥3.

### 2.9 Statistical analyses

We conducted a retrospective cohort study in both LTCFs. We calculated geometric mean concentrations of IgG, expressed in binding antibody units/ml, for SARS‐CoV‐2 spike glycoprotein (anti‐RBD and SFL IgG), and geometric mean titers of NAb to the different strains of virus. We compared the different IgG and NAb distributions between residents and HCWs using the Wilcoxon—Mann–Whitney test. The analyses were performed for noninfected vaccinated subjects from whom sera was collected ≥14 days after the last dose of vaccine received. When comparing proportions, we used the Fisher exact test. Samples with borderline NAb were considered positive in the comparisons between HCWs and residents. We analysed the data using Stata®17.0 software.

## 3 RESULTS

### 3.1 Attack rates (ARs) and description of the cohort

In LTCF‐1, where the epidemic was caused by an Alpha variant, 23% (5/22) of the exposed residents, all partially vaccinated, became infected. The AR was higher (9/19, 47%) in LTCF‐2, where 89% of the residents were twice vaccinated and the epidemic was caused by a Beta variant. In LTCF‐2, the AR was 50% (1/2) in the unvaccinated residents and 47% (8/17) in the twice vaccinated. According to the data provided by the local epidemiological teams, the AR in HCWs was 23% (7/31) in LTCF‐1 and 18% (6/34) in LTCF‐2. In LTCF‐2 the AR in twice vaccinated was 6% (1/16) whereas the AR in unvaccinated was 29% (5/17). Four residents died, and one resident was hospitalized during the outbreaks. All had tested positive for COVID‐19. One death and one hospitalization were ruled to be caused by COVID‐19, but it remained unclear whether the three other residents died with or because of COVID‐19. All other residents and HCWs had mild symptoms.

Description of the study population is presented in Table 1. The residents were aged at least 69 years and the majority were female (76%). There were no differences observed between the two LTCFs regarding the age, sex and comorbidity distribution of residents according to infection status (narrow case definition). The main comorbidities were dementia (88%), heart conditions (24%) and high blood pressure (64%). To identify potential additional cases and assess the extent of the outbreaks, we investigated the presence of antibodies indicative of past infection from serum samples collected from the subjects who participated in the seroepidemiological study. The serological analysis did not identify any previously undiagnosed cases (data not shown).

**Table 1:** Description of the study population, COVID‐19 outbreak in long‐term care facilities (LTCFs), 2021, Finland

	LTCF‐1		LTCF‐2	
	**PCR**+	**PCR−**	**PCR**+	**PCR−**
Number of residents	2	17	6	8
Number of HCWs	2	22	1	13
**Vaccination status, *n* (%)**				
Residents				
Unvaccinated	0	0	0	0
Vaccinated once	2 (100)	17 (100)	0	0
Vaccinated twice	0	0	6 (100)	8 (100)
HCWs				
Unvaccinated	2 (100)	6 (27)	1 (100)	3 (23)
Vaccinated once	0	12 (55)	0	0
Vaccinated twice	0	4 (18)	0	10 (77)
**Residents' description, *n* (%)**				
Median age in years (IQR)	87 (77–97)	83 (78–91)	82 (80–87)	82 (79–86)
Females	1 (50)	14 (82)	4 (67)	6 (75)
Comorbidities without dementia				
No comorbidities	0	5 (29)	3 (50)	1 (12)
1 comorbidity	2 (100)	8 (47)	1 (17)	4 (50)
≥2 comorbidities	0	4 (24)	2 (33)	3 (38)
**HCWs' description, *n* (%)**				
Median age in years (IQR)	34 (25–42)	35 (26–43)	36 (–)	41 (31–45)
Females	2 (100)	15 (68)	1 (100)	12 (92)
Comorbidities without dementia				
No comorbidities	2 (100)	17 (77)	1 (100)	11 (85)
1 comorbidity	0	4 (18)	0	2 (15)
≥2 comorbidities	0	1 (5)	0	0

Abbreviations: HCWs, healthcare workers, IQR, interquartile range.

### 3.2 COVID‐19 vaccinations of the residents and HCWs in the study cohort

The residents of LTCF‐1 were all partially vaccinated with Pfizer/BioNTech Comirnaty (Table 1). They had received their first vaccine dose at a median of 15 days (range: 15–19) before the first case was diagnosed. In the LTCF‐2, all residents who participated were vaccinated twice with Pfizer/BioNTech Comirnaty. They all received their second vaccine dose the same day, 27 days before the first case was diagnosed. The HCWs were vaccinated with either one dose of Pfizer/BioNTech Comirnaty (*n* = 8) or Oxford/AstraZeneca Vaxzervria (*n* = 4), or with two doses of Pfizer/BioNTech Comirnaty (*n* = 14). The once‐ and twice‐vaccinated HCWs had received their last vaccine dose at a median of 14 days (range: 7–20) and 31 (19–36) days, respectively, before the first case was diagnosed. The interval between doses was less than 6 weeks for both residents and HCWs.

We collected serum samples from the residents at a median of 36 days (range: 36–41 days) following their first COVID‐19 vaccine dose (LTCF‐1) or at a median of 54 days (range: 54–69 days) following their second vaccine dose (LTCF‐2). Two noninfected vaccinated HCWs had a sample collected less than 14 days after the last dose received. After the exclusion of these participants, the time from vaccination to sample collection among the HCWs was comparable to the residents; 33 days (range: 18–63 days) in once vaccinated and 63 days (20–78) in twice vaccinated. We collected PBMC samples from nine residents at 54 days and from three HCWs at 54–62 days following vaccination in LTCF‐2.

### 3.3 IgG antibody levels of vaccinated noninfected

**Figure 1:**
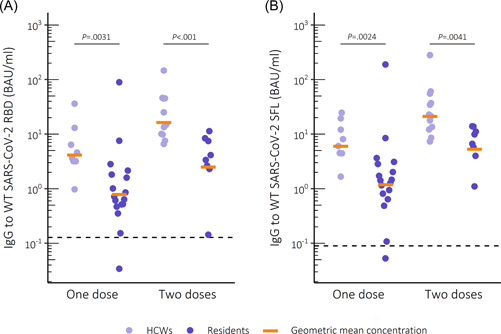
IgG levels to WT SARS‐CoV‐2 spike glycoprotein after vaccination with Comirnaty. (A) Receptor binding domain (RBD) and (B) full‐length spike glycoprotein (SFL) in binding antibody units (BAU)/ml. Dashed lines mark the threshold for positive result per antigen. Statistical significance measured with Wilcoxon rank‐sum test, significance level 0.05. IgG, immunoglobulin G; SARS‐CoV‐2, severe acute respiratory syndrome coronavirus 2.

We detected anti‐spike IgG among most (28/29) subjects who had received a single vaccine dose (Figure 1). However, the concentration of anti‐spike IgG was significantly higher in HCWs compared to residents (*p* = .0236 for RBD and *p* = .0109 for full‐length SFL), regardless of the type of vaccine received. In subjects who received one dose of Pfizer/BioNTech Comirnaty anti‐spike IgG levels were fivefold higher in HCWs compared to residents (*p* = .0031 for RBD, *p* = .0024 for SFL) (Table 2). Twice vaccinated HCWs had sixfold (RBD) and fourfold (SFL) higher anti‐spike IgG levels compared to twice vaccinated residents (*p* < .001 and *p* = .0041, respectively).

**Table 2:** Geometric mean neutralizing antibody (NAb) titers (GMT [95% CI]) to wild‐type (WT), Alpha and Beta variants of SARS‐CoV‐2 and anti‐spike IgG concentrations (GMC [95%CI]) expressed in binding antibody units/ml. Vaccinated and noninfected (PCR‐) with Pfizer/BioNTech Comirnaty, COVID‐19 outbreaks in long‐term care facilities (LTCFs), 2021, Finland

	Partially vaccinated (one dose)		Fully vaccinated (two doses)	
	Residents (*n* = 17)	HCWs (*n* = 8)	Residents (*n* = 8)	HCWs (*n* = 12)
NAb to WT	3.5 [2.0–6.3]^a^	8.8 [4.1–19]	18 [9.2–35]	110 [62–210]
NAb to Alpha variant	2.4 [1.6–3.7]^a^	3.4 [1.6–7.2]	5.3 [2.9–9.7]	39 [18–85]
NAb to Beta variant	2.1 [1.9–2.5]^a^	2.3 [1.7–3.2]	2.2 [1.8–2.7]	7.5 [3.8–15]
Anti‐RBD IgG	1.0 [0.41–2.3]	5.1 [2.1–12.6]	3.1 [1.0–9.7]	20 [11.3–37]
Anti‐SFL IgG	1.5 [0.60–3.6]	7.4 [3.6–15.5]	6.6 [3.2–13]	26 [14–50]

Abbreviations: CI, confidence interval; HCWs, healthcare workers, RBD, receptor binding domain, SFL, full‐length spike glycoprotein.

a No NAb measured for one resident due to limited sample volume.

### 3.4 NAb levels of vaccinated noninfected

**Figure 2:**
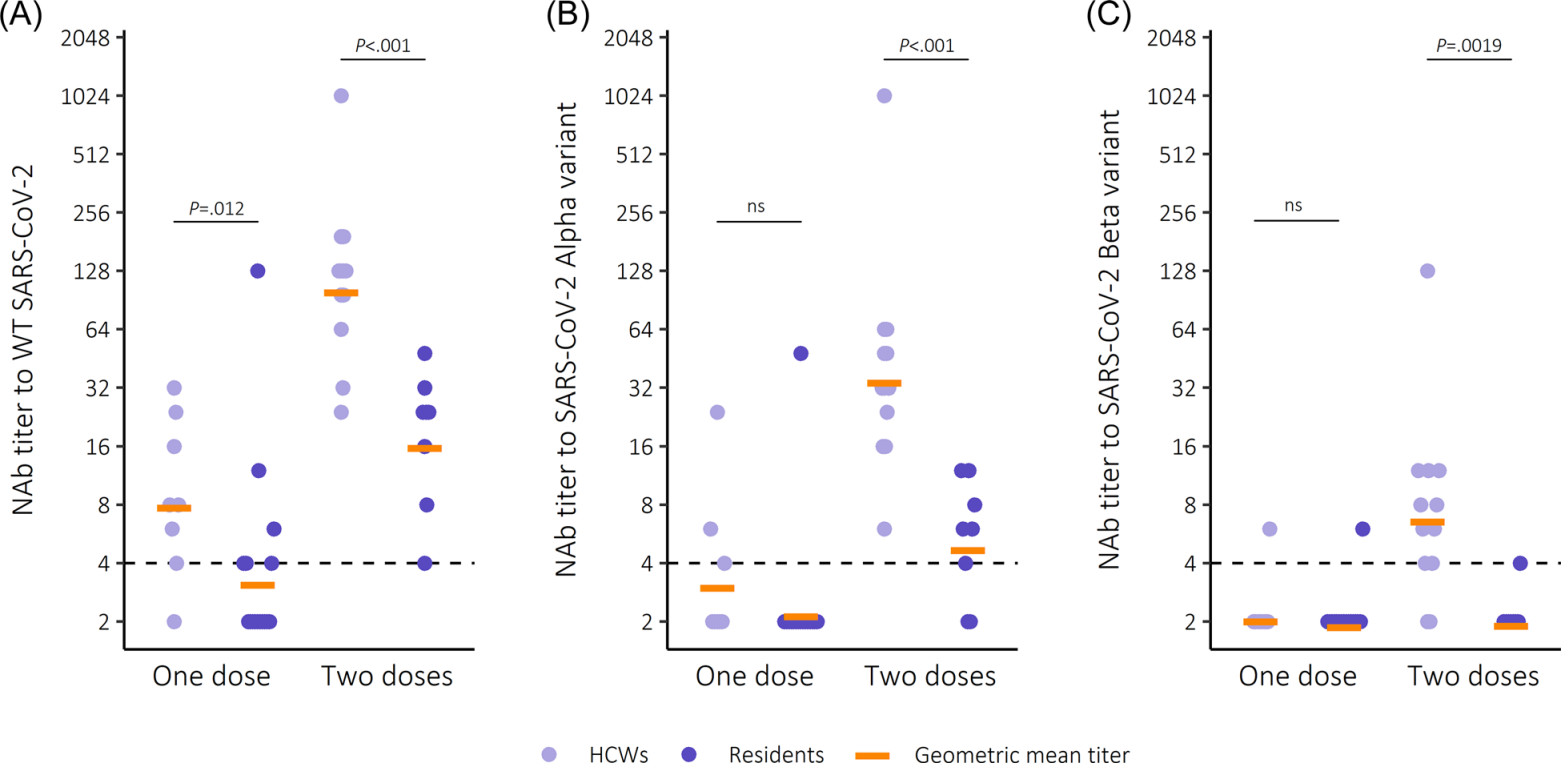
Neutralizing antibody (NAb) titers to SARS‐CoV‐2 (A) wild‐type (WT), (B) Alpha and (C) Beta variants after vaccination with Comirnaty. Dashed line marks the threshold for a positive NAb result (≥4). Statistical significance measured with Wilcoxon rank‐sum test, significance level 0.05. SARS‐CoV‐2, severe acute respiratory syndrome coronavirus.

The majority (9/12) of partially vaccinated, noninfected HCWs had NAb to WT SARS‐CoV‐2 in contrast to less than half of the residents (Figure 2). However, most partially vaccinated HCW and residents did not have NAb to Alpha and Beta variants (Figure 2). In subjects who had received one dose of Pfizer/BioNTech Comirnaty, NAb titers to WT SARS‐CoV‐2 were threefold higher in HCWs compared to residents (Table 2) (*p* = .0119), but there were no significant differences between the NAb titers to Alpha and Beta variants.

All twice vaccinated, noninfected HCWs and all residents had NAb to WT SARS‐CoV‐2 (Figure 2). All HCWs and 6/8 residents (including one borderline positive) had NAb to the Alpha variant. Of the HCW 10/12 had NAb against the Beta variant, including 2 borderline positives. In contrast, 7/8 residents had no NAb to the Beta variant, whilst 1 was borderline‐positive (Figure 2). In twice vaccinated noninfected, NAb titers to WT SARS‐CoV‐2 were sixfold higher in HCWs compared to residents (*p* < .001). NAb titers to the Alpha variant were sevenfold higher (*p* < .001) and NAb titers to the Beta variant were threefold higher (*p* = .0019) among HCWs (Table 2).

### 3.5 IgG antibody levels and NAb titers of twice vaccinated and infected versus noninfected

Although noninfected residents had lower antibody levels compared to HCWs, twice vaccinated and infected residents had high NAb titers and IgG concentrations (Figure S1 and Table S1). Twice vaccinated and infected residents had 27‐ and 35‐fold higher IgG concentrations to SFL and RBD, and 49‐, 58‐, and 54‐fold higher NAb titers to WT, Alpha and Beta variants, respectively, compared to twice vaccinated and noninfected residents (*p* < .001). Twice vaccinated and infected residents had also higher anti‐spike IgG concentrations (*p* = .018) and NAb titers to WT (*p* = .0039) Alpha (*p* = .047) and Beta (*p* = .0030) variants compared to twice vaccinated and noninfected HCWs.

### 3.6 Cell‐mediated immunity

**Figure 3:**
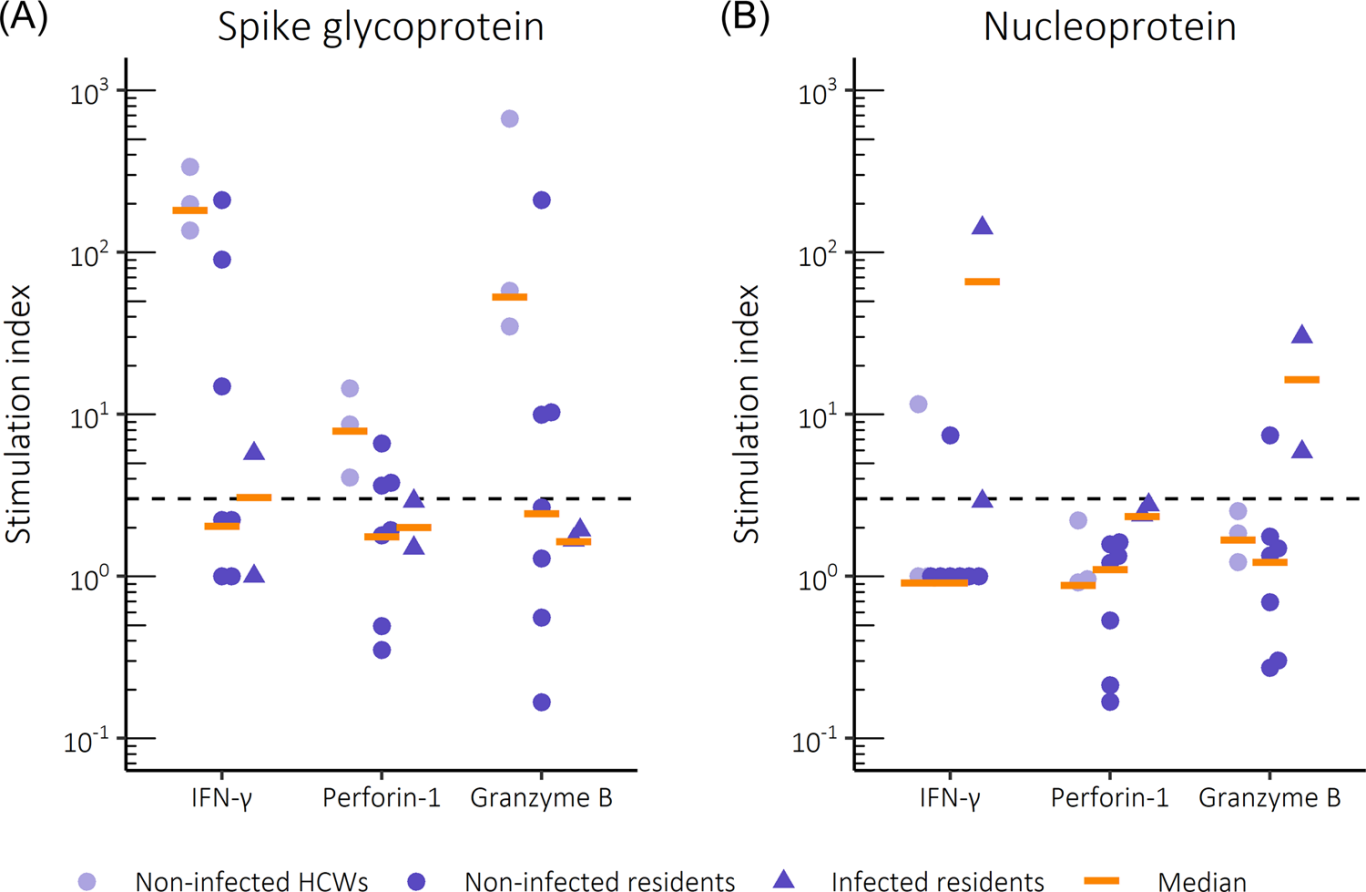
Peripheral blood mononuclear cell (PBMC) responses to stimulation with (A) SARS‐CoV‐2 recombinant spike glycoprotein or (B) SARS‐CoV‐2 recombinant nucleoprotein. The secretion of IFN‐γ, perforin‐1 or granzyme B into supernatants was measured after 6 days in infected or noninfected residents and healthcare workers (HCWs,) who all had received two doses of Comirnaty. Cytokine concentrations are presented as stimulation indexes. Dashed line marks the threshold for a positive result (stimulation index ≥3). IFN, interferon; SARS‐CoV‐2, severe acute respiratory syndrome coronavirus.

PBMCs of twice vaccinated and noninfected HCWs responded vigorously to stimulation with SARS‐CoV‐2 spike glycoprotein, with the secretion of IFN‐γ, granzyme B and perforin‐1 into supernatants. In comparison, only 3/7 samples from twice vaccinated and noninfected residents showed positive cellular responses to spike glycoprotein. Group‐level cellular responses directed at SARS‐CoV‐2 nucleoprotein remained low both in HCWs and residents, although one of each showed a positive response. In comparison, responses directed at SARS‐CoV‐2 nucleoprotein were strong in samples from two residents with positive antibody and PCR tests, consistent with breakthrough infection (Figure 3 and Figure S2).

## 4 DISCUSSION

Residents of the LTCFs investigated in this study remained susceptible to Alpha and Beta variant infections despite recent COVID‐19 vaccinations. In LTCF‐1, where the outbreak was caused by an Alpha variant, the AR was 23% among residents who had received a single dose of COVID‐19 vaccine 2 weeks prior. In LTCF‐2, the outbreak was caused by a Beta variant and 47% of exposed residents became infected despite receiving their second COVID‐19 vaccine dose 1 month before the outbreak. In the serological analysis we observed that the vaccinated residents had significantly lower levels of NAb and anti‐spike IgG compared to vaccinated HCWs, which likely contributes to the increased susceptibility of the elderly residents to breakthrough infections.

Our results and reports from other outbreaks in nursing homes^16^, ^17^ confirm the possibility of SARS‐CoV‐2 spread in nursing homes even when most residents are vaccinated. Others have reported that although vaccine effectiveness (VE) against infection by SARS‐CoV‐2 variants is remarkably decreased,^18^, ^19^, ^20^, ^21^ VE against severe illness remains high after two doses.^18^, ^21^, ^22^, ^23^ Among all infected residents in the two LTFCs four residents died after having tested positive for COVID‐19, but only one death was ruled to have been caused by COVID‐19 with certainty. Due to our small sample size and deaths with unclear relation to COVID‐19 we were unable to assess VE. A previous study reporting an outbreak caused by Alpha variant in an elderly nursing home in France found that while vaccination did not prevent the outbreak, it reduced disease severity.^24^ Although our sample size was limited, considering one death and one hospitalization despite the high proportion of comorbidities among the residents, this was likely also the case in the two LTCFs studied.

We found that LTCF residents had significantly lower antibody levels following vaccination when compared to HCWs. A recent meta‐analysis found that higher NAb titers correlate strongly with protection from symptomatic infection caused by different variants of concern.^25^ Other studies have also reported that the elderly have lower NAb titers compared to HCWs, regardless of variant and number of COVID‐19 vaccine doses received.^26^, ^27^ Only 1/8 of the twice vaccinated residents in our study had NAb to Beta variant even though we used a highly sensitive microneutralization test.^11^, ^12^ Our results also demonstrated reduced cellular responses against SARS‐CoV‐2 spike in vaccinated elderly residents compared to vaccinated HCW. Our findings on lower serum neutralization potency and reduced cellular responses among the elderly are consistent with previous reports.^26^, ^28^ The IgG and NAb levels among PCR‐positive, previously vaccinated elderly subjects were markedly elevated. Other studies have also reported the boosting effect of breakthrough infections on vaccine‐primed antibody responses.^29^ Our findings thus indicate that despite weaker vaccine responses, elderly subjects can mount markedly stronger responses following exposure.

The main limitation of our study was the small sample size. The nature of the study and the LTCFs studied intrinsically limited the possible number of participants, but we also encountered a very low participation rate of HCWs. Only 3/13 HCWs who tested PCR‐positive during the outbreaks participated. Furthermore, we were able to only compare the immune responses of those that had received one or two doses of Comirnaty, as our sample size did not allow comparison of other vaccine products. One of the strengths of our study was that by combining the epidemiological investigation and PCR‐test results with the assessment of serological signs of infection, we could get a more reliable estimate of ARs among the participants. The serological survey confirmed that all cases in this study cohort had been identified by PCR screening during the initial epidemiological investigation. Moreover, by collecting samples from both HCWs and the elderly residents in a setting where both groups had been vaccinated with similar schedules, we could identify significant differences in the humoral and cellular immune responses to vaccination between these two groups.

Outbreaks in nursing homes continue to occur even in care homes where most residents are vaccinated. Here we report that in a LTCF outbreak caused by the Beta variant, AR was 47% in a twice vaccinated population, and that NAb to the Beta variant were only detected from 1/8 of the twice vaccinated residents. The weaker response of the elderly to the COVID‐19 vaccines is accentuated in a situation where the viruses causing the outbreaks are capable of evading immunity and only a part of the antibodies targeting the vaccine‐type spike protein can neutralize the viral variants. In a recent study we conducted in another nursing home, we observed that while 7/7 nursing home residents had NAb to Delta, 2/7 did not have measurable NAb to either Beta or Omicron variant despite receiving a Pfizer/BioNTech Comirnaty booster 21–42 days prior.^27^ These are alarming findings and speak of the need for new solutions, e.g., vaccine formulations, to best protect the frail and elderly in this pandemic.

## AUTHOR CONTRIBUTIONS

**Dorothée Obach, Anna Solastie, Oona Liedes, Saimi Vara, Eva Krzyżewska‐Dudek, Tobias L. Freitag, Charlotte C. Hammer and Merit Melin**: analysed the results. **Dorothée Obach, Anna Solastie, Charlotte C. Hammer, Timothée Dub, Outi Lyytikäinen, Tobias L. Freitag, Seppo Meri and Merit Melin**: designed the study. **Luise Brinkmann, Oona Liedes, Saimi Vara, Eva Krzyżewska‐Dudek and Anu Haveri**: performed laboratory analyses. **Dorothée Obach and Anna Solastie**: wrote the paper with input from all authors. All authors approved the final version.

## CONFLICTS OF INTEREST

The Finnish Institute for Health and Welfare has received research funding for studies not related to COVID‐19 from GlaxoSmithKline Vaccines, in which Merit Melin was an investigator. Tobias L. Freitag is an employee of Rokote Laboratories Finland Oy, that is developing a nasal vaccine against SARS‐CoV‐2. Rokote Laboratories Finland has no role in this study. The other authors declare no conflicts of interest.

## ETHICS STATEMENT

The Finnish communicable diseases law^30^ and the law on the duties of the Finnish Institute for Health and Welfare^31^ allowed the implementation of the epidemiological investigation without seeking further institutional ethical review. Participation of the residents and HCW was voluntary and written information on the investigation was given before participation.

## Supporting information

Supporting information.

## Data Availability

Data and code are available from corresponding authors upon reasonable request.
