# Supplementary material for: Impaired immunity and high attack rates caused by SARS‐CoV‐2 variants among vaccinated long‐term care facility residents
Source: Immun Inflamm Dis. 2022 Aug 17;10(9):e679. doi: 10.1002/iid3.679 (PMC9382858; doi:10.1002/iid3.679)
Supplement: Supplementary file 1 — Supporting information. [file IID3-10-0-s001.docx]

Supplementary material


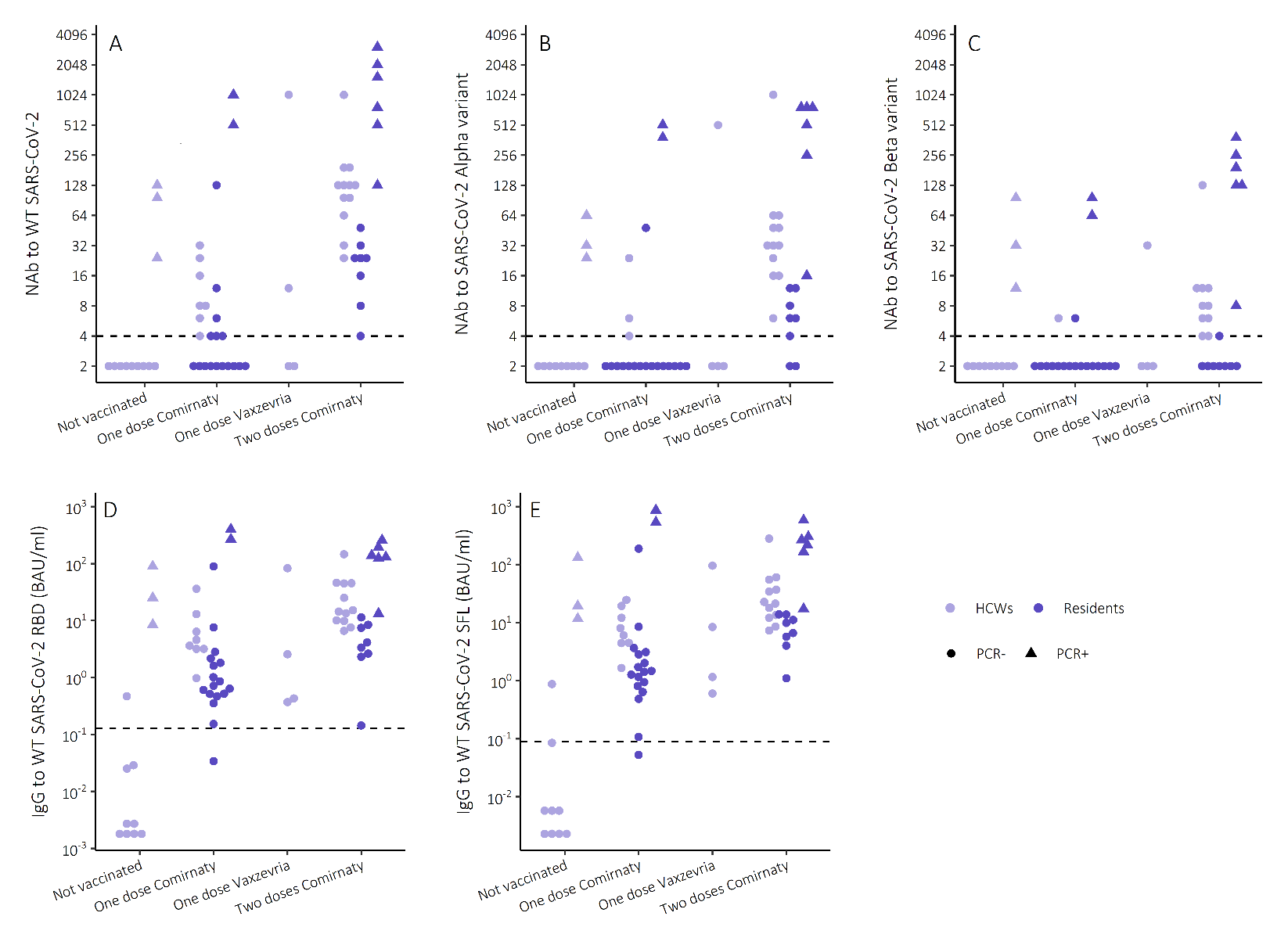


Figure S1. Neutralizing antibody (NAb) and anti-spike IgG levels in study participants. A) NAb to wild-type (WT) SARS-CoV-2. B) NAb to SARS-CoV-2 Alpha variant. C) NAb to SARS-CoV-2 Beta variant. D) IgG to receptor binding domain (RBD) of WT SARS-CoV-2. E. IgG to full-length spike glycoprotein (SFL) of WT SARS-CoV-2. IgG levels presented as binding antibody units (BAU/ml). Dashed lines indicate threshold for positive result.


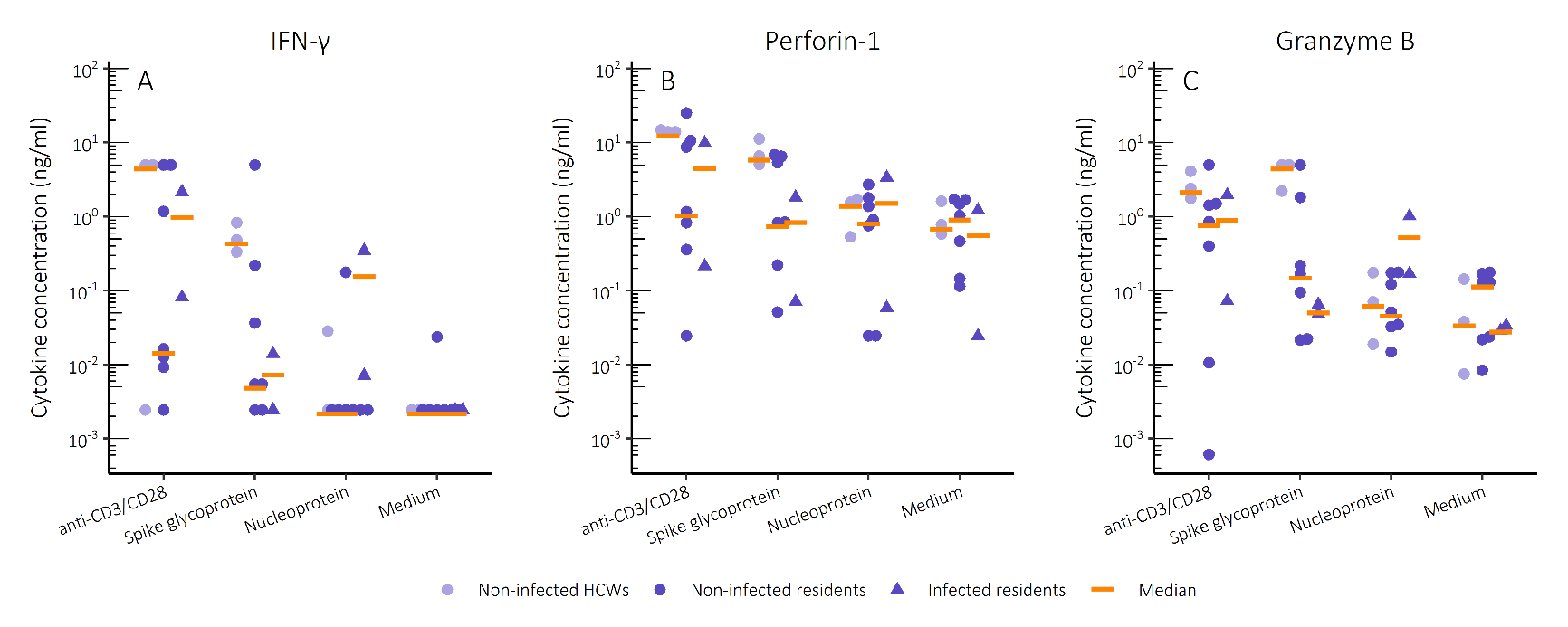


Figure S2. Peripheral blood mononuclear cell (PBMC) responses to stimulation with positive control (anti-CD3/CD28), SARS-CoV-2 recombinant spike glycoprotein, nucleoprotein or medium in infected or non-infected residents and healthcare workers (HCWs) that all had received two doses of Comirnaty. The secretion of A) IFN-γ, B) perforin-1 or C) granzyme B into supernatants was measured after 6 days.

Table S1. Geometric mean IgG concentrations (GMC [95% confidence interval]) and neutralizing antibody (NAb) titers (GMT [95%CI]) in infected and non-infected study participants that had received two doses of Comirnaty.

|  | Infected, twice vaccinated residents  n=6 | Non-infected, twice vaccinated residents  n=8 | Non-infected, twice vaccinated HCWs  n=12 |
| --- | --- | --- | --- |
| Anti-RBD IgG | 110 [35–330] | 3.1 [0.99–9.7] | 20 [11–37] |
| Anti-SFL IgG | 180 [49–630] | 6.6 [3.2–13] | 26 [14–50] |
| NAb to WT | 890 [270–3000] | 18 [9.2–35] | 110 [62–210] |
| NAb to Alpha variant | 310 [64–1500] | 5.3 [2.9–9,7] | 39 [18–85] |
| NAb to Beta variant | 120 [27–490] | 2.2 [1.8–2.7] | 7.5 [3.8–15] |

HCWs = healthcare workers, RBD = receptor binding domain, SFL = full-length spike glycoprotein, WT = wild-type, GMC = geometric mean concentration, expressed in binding antibody units (BAU/ml), GMT = geometric mean titer. CI = confidence interval.
